# A Rare Case of a Large Primary Renal Neuroendocrine Tumour: A Case Report and Brief Review of Literature

**DOI:** 10.7759/cureus.19743

**Published:** 2021-11-19

**Authors:** Matthew J Deacon, Hannah Harvey, Chirag Shah, Azhar Khan

**Affiliations:** 1 Urology Department, King's College Hospital, London, GBR; 2 Pathology Department, King's College Hospital, London, GBR

**Keywords:** immunohistochemistry, somatostatin analogues, cancer metastasis, open nephrectomy, renal tumour, neuroendocrine tumour

## Abstract

Primary renal neuroendocrine tumours are very rare clinical entities, and as such, relatively little is known about their clinical progression. Here, we outline the case of a young female patient presenting with abdominal pain and a large 14 cm right renal mass. Initial radiological studies demonstrated localised disease, but during surgical resection, widespread liver metastasis was identified. Histological analysis revealed a grade 2, well-differentiated neuroendocrine tumour pT3a. Whilst surgical resection remains the gold standard for localised disease, further work is required to understand the pathogenesis, prognostic indicators and treatment following metastatic spread. The poor prognosis seen in primary renal neuroendocrine neoplasia highlights the importance of further directed research in this area.

## Introduction

Neuroendocrine neoplasms (NENs) are a rare group of tumours arising from the neuroendocrine cell system and are most commonly found to affect the gastrointestinal tract and lungs [1,2]. Primary neuroendocrine tumours of the kidney are a very rare subtype of these neoplasms, with less than 100 cases described in the literature [3-6]. The World Health Organization (WHO) categorises renal NENs (formerly known as carcinoid of the kidney) into well-differentiated neuroendocrine tumours, large cell neuroendocrine carcinoma (NEC), small cell NEC and phaeochromocytoma [7]. Immunohistochemical features include expressions for synaptophysin, chromogranin A and CD56, although individual features can be highly variable [6,8]. The prognosis of primary renal NEN is generally poor, with a five-year survival estimated around 50%, although a large variation within this group is seen [4,6,9]. The rarity of these cases and the paucity of published literature mean that much regarding primary renal neuroendocrine neoplasms is still to be understood.

Here, we outline the case of a young female patient presenting with a well-differentiated primary renal neuroendocrine tumour and subsequent metastasis.

## Case presentation

A 36-year-old female presented to the urology department with a two-month history of right-sided abdominal pain and backache. She denied haematuria or any other urinary or gastrointestinal symptoms. On retrospective review, she admitted to experiencing skin flushing once a week and palpitations and hot sweats but denied diarrhoeal symptoms. She was otherwise fit and well, and her only past medical history was of long-standing migraines for which she took zolmitriptan. She had never smoked and had no family history of renal or other neuroendocrine tumours. On examination, she had a palpable and minimally tender right-sided mass.

**Figure 1:**
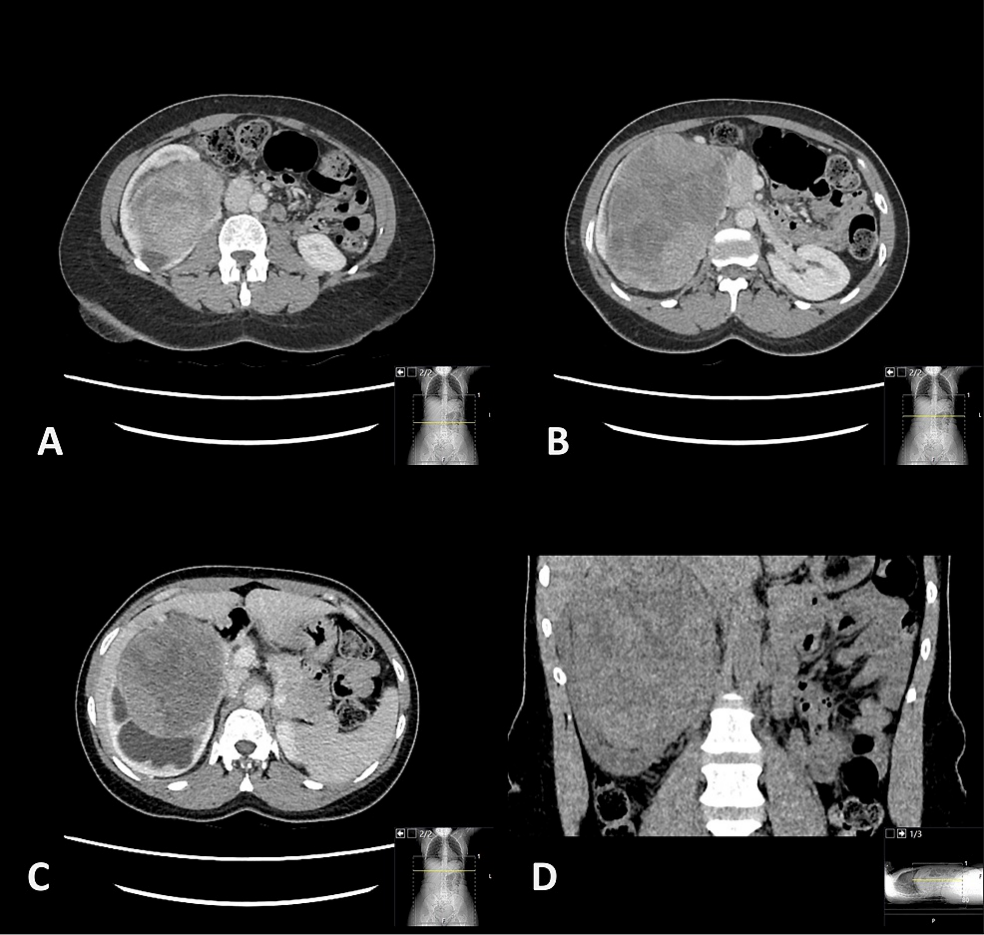
Pre-operative CT images demonstrating the large 14 cm right renal mass. (A-C) Axial and (D) coronal slices are shown. The liver was reported as clear of metastasis on CT. CT: computed tomography

An ultrasound and subsequent computed tomography (CT) scan were arranged, which revealed a 14 cm mass arising from the upper pole of the right kidney (Fig. 1). The tumour was abutting, but not invading the liver, and no evidence of metastatic spread was seen, either in the liver or elsewhere. Pre-operative blood workup revealed normal renal and liver function and mild normochromic anaemia.

The patient then underwent an open right radical nephrectomy. The operative findings were of a large 14 cm right upper pole tumour abutting the liver but with a clear plane between the liver and the kidney. After the removal of the right kidney with tumour, the liver was inspected and found to have more than 10 small lesions throughout the anterior surface occupying all of the visible liver segments. Most were less than 5 mm in diameter, but one was larger, measuring 3 cm in size. One liver lesion was sent for fresh frozen section, and an intra-operative diagnosis of liver metastasis was made. Owing to the large number of metastasis and their distribution throughout the liver, they were felt to be unresectable, and the operation was concluded with only the right radical nephrectomy performed.

Post-operatively, the patient made a good recovery and was discharged after five days in the hospital without significant complications.

Histopathological examination of the specimen found that the renal parenchyma had been largely replaced by a 144 mm haemorrhagic tumour displaying solid and cystic areas. The anatomy of the hilum had been grossly distorted, and foci suspicious for necrosis were seen.

**Figure 2:**
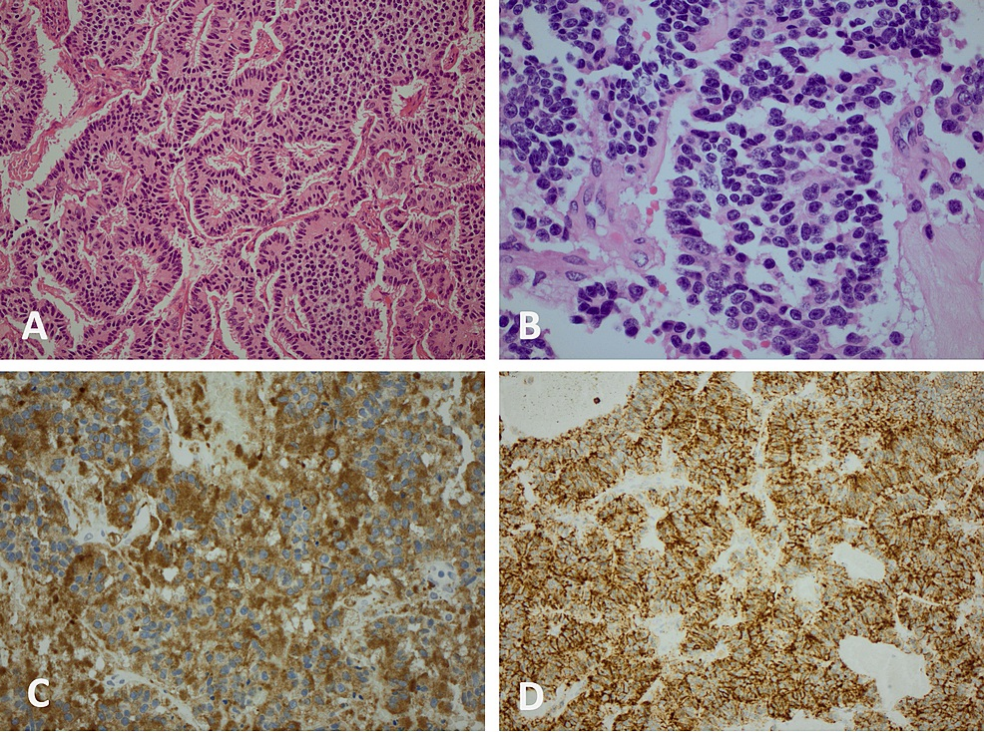
Histological slides of the operative specimen confirming a well-differentiated neuroendocrine tumour of the right kidney. (A) HE 20× highlighting trabecular and rosette-like patterns. (B) HE 60× demonstrating stippled (salt-and-pepper) chromatin. (C) Positive staining with synaptophysin immunostain. (D) Positive staining with CD56 immunostain. HE: haematoxylin and eosin stain

Microscopy revealed tumour cells arranged in trabeculae, nests and ribbons, with focal attempts to form rosette-like structures (Fig. 2A). The tumour cells were monomorphic round to polygonal, with granular amphophilic to eosinophilic cytoplasm and uniform round nuclei with stippled (salt-and-pepper) chromatin (Fig. 2B). Immunohistochemistry was performed and showed tumour cells staining positive for synaptophysin (Fig. 2C), CD56 (Fig. 2D), CD57, chromogranin (focal) and alpha-methylacyl-CoA-racemase (AMACR) (weak). Staining with CK7, CD10, PAX8, Melan A, HMB45 and WT1 are all negative. The proliferation index (Ki-67) was 5%. The mitotic count was 1 per 10 HPF. The tumour was focally invading the wall of the renal capsule, but extra-capsular spread was not seen. At the hilum, the tumour appeared to have obliterated the normal sinus architecture; the tumour was seen within severely disrupted vascular structures with fragmented elastic fibres. Peri-capsular and intra-tumoural lymphovascular invasion was also noted.

Overall, the morphological and immunohistochemical features were in keeping with a grade 2 well-differentiated neuroendocrine tumour pT3a. Further examination of the liver metastasis confirmed a grade 2 neuroendocrine tumour, with immunohistochemistry showing strong diffuse positivity with synaptophysin and CD56, and ISL-1 was also positive. The proliferation rate in “hot spots” was 5.9%.

Two months post-operatively, a Gallium-68 DOTATATE positron emission tomography (PET) scan was performed, which confirmed bilobar liver and pelvic skeletal metastases with variable somatostatin receptor expression. No alternative primary malignant site was identified. Subsequent blood tests for levels of gastrin, somatostatin, vasoactive intestinal peptide and chromogranin A, as well as a 24-hour urinary metanephrine collection, have all been normal.

She has now been started on monthly intravenous somatostatin analogue injections, which will be continued for six months. Clinically, she is experiencing gastrointestinal side effects related to these injections but is otherwise clinically stable at six months post-surgery. She will continue close follow-up within a specialist neuroendocrine tumour clinic with three-monthly blood tests and clinical review, and after six months of somatostatin analogue treatment, a follow-up PET scan and MRI liver will be performed to assess radiological response.

## Discussion

Neuroendocrine neoplasms are very uncommon tumours in general, with an age-adjusted incidence in western populations of around 8 per 100,000 across all NEN types [1,2]. The vast majority of these affect the gastrointestinal tract or lungs, with primary neuroendocrine tumours of the kidney making up less than 0.4% of all NENs [10]. Primary renal NENs form just a tiny fraction of all primary renal tumours, estimated at less than 0.3% [11]. Analysis of the SEER registry by McGarrah et al. suggested that the incidence of primary renal NENs is 0.13 per one million persons [5].

Perhaps owing to their rarity, the pathophysiology of NENs is not completely understood. Cells of the neuroendocrine system are not usually present within the kidney, but one theory is that tumours could arise from the activation of aberrant gene sequences of multipotent stem cells, which are common to those of neuroendocrine differentiated cells [12,13]. Other theories regarding the origin of abnormal neuroendocrine cells within the kidney include metaplasia of the pyelocaliceal urothelium by chronic inflammation, metastasis from undiscovered primary tumours or entrapped neural crest tissue in the kidney during embryogenesis [13]. An association of NENs forming within abnormal renal tracts, such as horseshoe kidneys, has been described [3,5,13], but of note, the case that we describe here was of a tumour in an otherwise normal kidney with a normal contralateral kidney in situ.

The clinical presentation of renal NENs tends to be similar to that of the more common primary renal tumours. A significant number present asymptomatically, but for those with symptoms, flank or abdominal pain (with or without haematuria) are the most common [6,13,11]. Presentation with carcinoid syndrome or features of neuroendocrine crisis is rare, and most renal NENs are not found to be biochemically active [5,12]. On retrospective questioning, our patient did report some mild flushing episodes prior to diagnosis, but no diarrhoeal symptoms, and haematuria was not present despite the obliteration of the normal sinus architecture on microscopy.

Another interesting feature of our case was the finding of liver metastases intra-operatively despite no radiographic evidence on CT scan just a short number of weeks previous. Subsequent MRI and DOTATATE PET scanning showed both liver and skeletal metastases. No radiological features on CT entirely specific to renal NENs are described, although calcification is often noted [6,13]. Gallium-68-based PET scanning has widespread use in the follow-up of patients with NETs of other sites [14] and can be useful in patients with known renal NEN, particularly in the search for disseminated metastasis.

The immunohistochemical features of primary renal NENs include cells that stain positive for synaptophysin, chromogranin A and CD56 [4,6,8]. These cells do not stain positive for other markers typical of renal cell carcinomas, such as WT-1 and AMACR [4], and are negative for TTF1, CK7 and CK20, which rule out the lung and bowel as primary sources [6]. Histological features can be more heterogeneous and less specific to renal neuroendocrine neoplasia, but those described include trabecular and rosette-like architectural patterns mixed with nests and cords of cells, palisading and tubal formation, and round nuclei with stippled (salt-and-pepper) chromatin [4,6]. The proposed significant adverse prognostic factors include age > 40 years, tumour size > 4 cm, purely solid tumours on the cut surface, a mitotic rate higher than 1/10 HPF, metastasis at initial diagnosis and tumours extending throughout the renal capsule [13,15]. Unfortunately, several of these poor prognostic indicators were present in the case presented here.

Surgical resection with either partial or radical nephrectomy makes up the mainstay for localised disease, but where metastasis is present, either pre- or post-operatively, then systemic therapies for renal NENs have been directed by the treatment of non-renal NENs. Somatostatin analogues such as octreotide, platinum-based chemotherapies and monoclonal antibodies have all been described [4-6,13]. Post-operatively, our patient has been established on monthly somatostatin analogue injections and is tolerating treatment well at the six-month stage.

## Conclusions

In conclusion, we present the rare case of a large, well-differentiated, primary renal neuroendocrine tumour in a young female patient. Although appearing to be localised on imaging, the operative findings revealed widespread liver metastasis, which was confirmed on frozen section and demonstrated clearly on post-operative PET scanning. Our patient has now been established on systemic somatostatin analogues and is doing well at six months post-operation.

The case highlights the importance of considering primary renal NEN in both the workup and histological evaluation of renal tumours and contributes further evidence to the understanding of this rare clinical entity.
